# Enhancing vaccine antibody responses by targeting Clec9A on dendritic cells

**DOI:** 10.1038/s41541-017-0033-5

**Published:** 2017-11-06

**Authors:** Hae-Young Park, Peck S. Tan, Ranmali Kavishna, Anna Ker, Jinhua Lu, Conrad E. Z. Chan, Brendon J. Hanson, Paul A. MacAry, Irina Caminschi, Ken Shortman, Sylvie Alonso, Mireille H. Lahoud

**Affiliations:** 1 Infection and Immunity Program, Monash Biomedicine Discovery Institute and Department of Biochemistry and Molecular Biology, Monash University, Melbourne, VIC Australia; 2 Centre for Biomedical Research, Burnet Institute, Melbourne, VIC Australia; 3 Department of Microbiology and Immunology, Yong Loo Lin School of Medicine, and Immunology Programme, Life Sciences Institute, National University of Singapore, Singapore, Singapore; 4 DSO National Laboratories, Singapore, Singapore; 5 Department of Microbiology and Immunology, The University of Melbourne, at the Peter Doherty Institute for Infection and Immunity, Melbourne, VIC Australia; 6 The Walter and Eliza Hall Institute, Melbourne, VIC Australia; 7 Department of Medical Biology, University of Melbourne, Melbourne, VIC Australia

## Abstract

Targeting model antigens (Ags) to Clec9A on DC has been shown to induce, not only cytotoxic T cells, but also high levels of Ab. In fact, Ab responses against immunogenic Ag were effectively generated even in the absence of DC-activating adjuvants. Here we tested if targeting weakly immunogenic putative subunit vaccine Ags to Clec9A could enhance Ab responses to a level likely to be protective. The proposed “universal” influenza Ag, M2e and the enterovirus 71 Ag, SP70 were linked to anti-Clec9A Abs and injected into mice. Targeting these Ags to Clec9A greatly increased Ab titres. For optimal responses, a DC-activating adjuvant was required. For optimal responses, a boost injection was also needed, but the high Ab titres against the targeting construct blocked Clec9A-targeted boosting. Heterologous prime-boost strategies avoiding cross-reactivity between the priming and boosting targeting constructs overcame this limitation. In addition, targeting small amounts of Ag to Clec9A served as an efficient priming for a conventional boost with higher levels of untargeted Ag. Using this Clec9A-targeted priming, conventional boosting strategy, M2e immunisation protected mice from infection with lethal doses of influenza H1N1 virus.

## Vaccine technology: targeting the vaccine to the immune system

Duration and intensity of vaccine response can be boosted using antibodies to target pathogen fragments to specific immune system cells. Dendritic cells exist to take fragments of infectious diseases and present them to the immune system, sparking host defenses. Now, researchers led by Monash University’s Mireille Lahoud, and Ken Shortman of the Walter and Eliza Hall Institute, have successfully used antibodies to target fragments of influenza and hand, foot and mouth disease directly to dendritic cell molecules, specifically chosen to elicit a prolonged immune response. Mice inoculated with the targeted vaccine were protected from lethal influenza exposure, whereas the hand, foot and mouth disease vaccine elicited promising, but less marked results. With further development, this technology could provide a vital boost to vaccines that offer poor immunity on their own.

## Introduction

Dendritic cells (DCs), as the key antigen (Ag)-presenting cells, are a logical target for immune response modulation, including improving the response to vaccines. One approach is to target the normal DC network in situ by injecting putative vaccines molecules coupled to a mAb recognising a DC surface molecule.^1–4^ By choosing a DC subset-specific mAb, it is possible to restrict initial Ag presentation to a particular DC subtype and so tailor the immune response. This approach generally involves co-injection of an adjuvant or DC activation agent, to ensure the presenting DC initiates an effective immune response rather than tolerance. Clinical trials have been undertaken, targeting human immunodeficiency virus and tumour Ag to the DC surface molecule DEC205.^5,6^

Another promising DC surface target is the C-type lectin-like receptor Clec9A, also termed DNGR1.^7–9^ This receptor is specifically expressed along with XCR1 by a DC subtype common to mouse and humans, recently termed conventional DC1 (cDC1).^10–12^ This subtype includes the CD8^+^ mouse DC lineage and its migratory CD103^+^ equivalent, and the human CD141^+^ DCs. This DC subtype is especially efficient at taking up and processing Ag from dead cells, and cross-presenting these Ag on major histocompatibility complex (MHC) class I. Clec9A is a receptor involved in this process, binding filamentous actin exposed when the cell membrane is damaged and facilitating the cross-presentation of dead cell-associated Ag.^13,14^ Mice lacking Clec9A are more susceptible to viral infections.^15^ Thus, targeting vaccine Ags to Clec9A plugs them into a natural and efficient Ag uptake and processing system.

The nature of the immune response on targeting Ag to Clec9A has been studied in mice using model Ags such as ovalbumin (OVA) and nitrophenol (NP).^7,9,16–18^ As expected, excellent cross-presentation of Ag on MHC class I was obtained, leading to an effective cytotoxic T-cell (CTL) response in the presence of DC activation agents. A surprising finding was that efficient Ag presentation on MHC class II was also obtained, leading to a remarkably high Ab response with a single injection of tiny doses of Ag. Equally surprising was the finding that such a high Ab response could be obtained in the absence of adjuvants and any sign of initial DC activation. Such Ab responses on targeting Clec9A were much higher than those obtained by targeting some other DC surface molecules including DEC205.^17^ Similar high Ab responses were obtained targeting Clec9A in macaques^19^ and similar high T-cell responses on targeting CLEC9A on human DC in humanised mice,^20^ suggesting effective translation to the human immune system should be possible.

The basis of the high Ab response has been explored in detail.^16–19,21^ As Clec9A is restricted in expression to rare DCs, the injected anti-Clec9A-Ag construct is not immediately absorbed but persists in the circulation. This leads to prolonged Ag presentation even by non-activated DCs, driving extensive and prolonged follicular helper T-cell (T_FH_) generation and memory. The final result is a high and persistent Ab response, involving germinal centre formation and affinity maturation, even in the absence of adjuvants. Thus, Clec9A targeting has considerable promise for generation of protective Ab responses to infectious diseases, as well as for CTL responses against tumours.

It was important to assess if these promising findings with model Ags would also apply to infectious disease vaccine Ags. Accordingly we selected two pathogen-derived subunit vaccine candidate molecules for targeting to mouse Clec9A. The first vaccine candidate was the ectodomain of the matrix protein 2 (M2e) from influenza that has been remarkably conserved in all human influenza strains over the past 80 years.^22,23^ We used a consensus M2e sequence that has the potential to confer protection against a wide range of influenza virus strains including the latest H1N1 2009 pandemic strain.^23^ Importantly, we have a mouse infection model for testing the efficacy of our M2e vaccine.^24^ The second vaccine candidate was SP70 from the hand, foot and mouth disease enterovirus 71 (EV71), a 15-amino-acid peptide from the capsid protein VP1 that is highly conserved among EV71 genogroups and subgenogroups.^25,26^ SP70 contains a B-cell neutralising epitope but no T epitopes.^25^ Both candidate vaccine Ags are poorly immunogenic alone.^23,26^ Thus, we aimed to deliver these Ags to DC using our Clec9A targeting constructs and determine whether our immunisation strategy could enhance the responses to a level that might be protective. In view of the side effects often associated with adjuvants, we also assessed if DC-activating agents would be essential for such a response. Finally, we tested whether a second administration of the vaccine could boost the primary Ab responses. Our findings demonstrate that targeting weakly immunogenic vaccine Ags to Clec9A markedly increases Ab responses. Furthermore, we show that targeting a “universal” influenza Ag to Clec9A, as a priming step in a heterologous prime-boost strategy, leads to strong protection from influenza infection. Our results provide many points of guidance for the application of DC-targeted vaccines to human populations.

## Results

### Initial targeting constructs

**Fig. 1:**
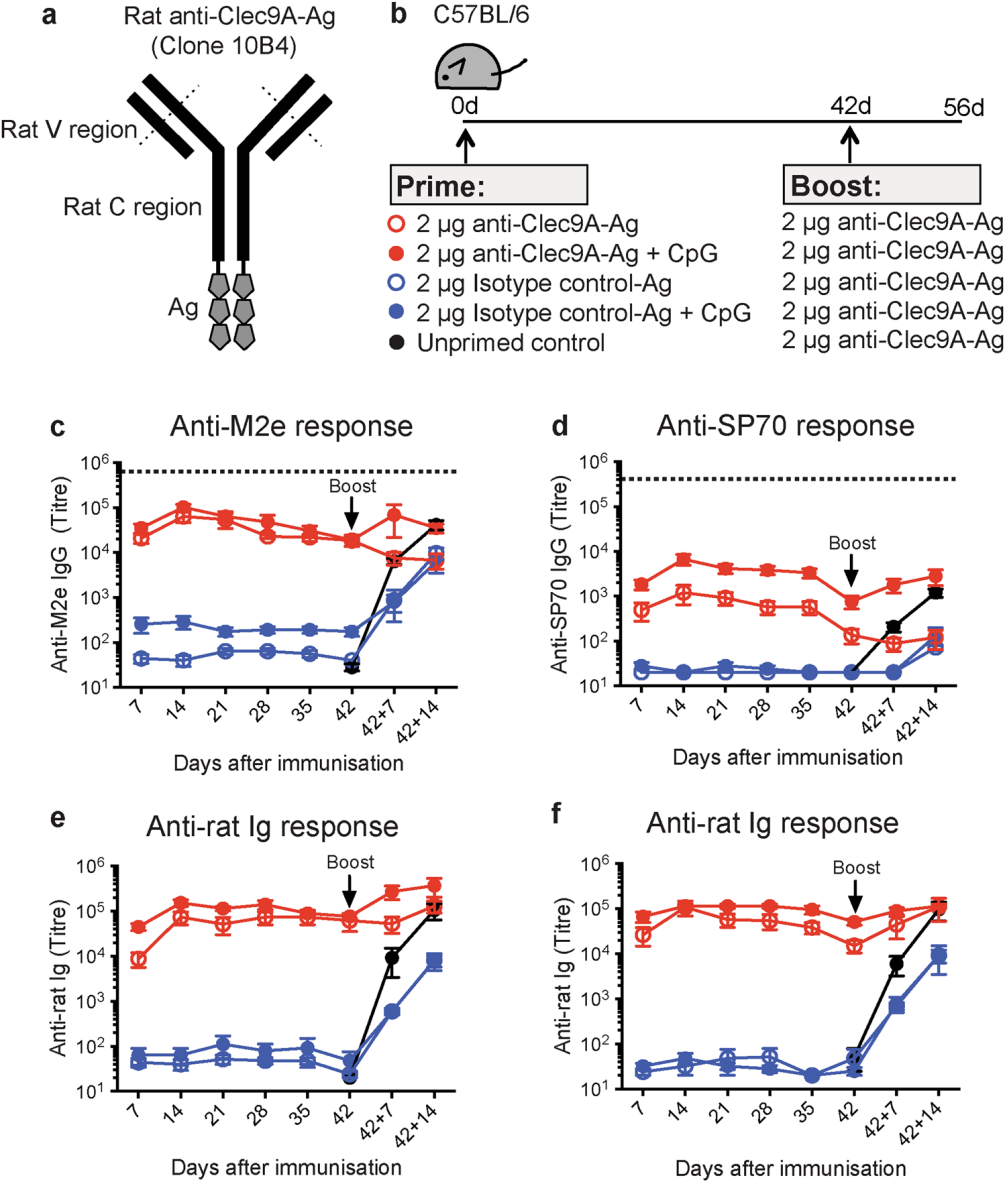
Targeting vaccine Ags to Clec9A generates long-lasting enhanced Ab responses. **a** Schematic representation of rat anti-Clec9A-Ag 10B4 constructs **b** Schematic representation of the vaccination strategy. C57BL/6 mice were injected i.v. with 2 μg anti-Clec9A-M2e mAb construct (clone 10B4), 2 μg isotype control-M2e mAb construct (clone GL117), 2 μg anti-Clec9A-SP70 mAb construct, or 2 μg isotype control-SP70 mAb construct in the absence or presence of adjuvant, 5 nmol CpG-1668. A group of mice were injected with PBS as unprimed control. At day 42 after priming, all groups of mice were boosted with 2 μg anti-Clec9A-M2e mAb construct or with 2 μg anti-Clec9A-SP70 mAb construct. Serum samples were collected at the indicated times post immunisation and Ag-specific IgG responses measured by ELISA. **c** Targeted versus (vs.) untargeted anti-M2e responses. **d** Targeted vs. untargeted anti-SP70 responses. **e** The anti-rat Ig response associated with c. **f** The anti-rat Ig response associated with d. Each point represents the geometric mean end point titre with 95% confidence interval (CI) from a group of five mice. The dotted line in c and d represents a “maximal” response obtained in separate experiments by injection of C57BL/6 mice with 50 μg KLH-Ag in Freund’s complete adjuvant followed by boosting with 50 μg KLH-Ag in Freund’s incomplete adjuvant and sampling the blood 30 days later. This full kinetic experiment was carried out once (*n* = 5 mice per group) but the results were confirmed at restricted time points in two other experiments

To deliver the putative vaccine Ags to Clec9A on DCs, we first generated constructs where three copies of M2e or SP70 in tandem were genetically fused to the Fc of the heavy chain of the rat IgG2a anti-Clec9A mAb 10B4,^7^ as illustrated in Fig. 1a. This mimicked the effective constructs previously used to target OVA to Clec9A.^17^ Non-targeting control constructs were also prepared, with the Ags fused to an isotype-matched control mAb GL117^17^ that does not bind DC. As in previous studies,^17,19^ we verified that the putative targeting constructs retained their ability to bind Clec9A on the cell surface. Since in our previous studies, injection of the B-cell hapten NP conjugated to 10B4 into C57BL/6 mice gave excellent Ab responses to NP,^16^ it was considered that the rat IgG2a of the construct provided the necessary T-cell epitopes for an Ab response to the B-cell epitopes of SP70. The rat Ig of the mAb construct also provided an internal control for effective Clec9A targeting after injection, since as shown previously a strong anti-rat Ig Ab response should be induced.^2,19^

### Ab responses to single injections of the targeted Ags

In preliminary experiments to determine the optimal delivery route, we immunised mice with the anti-Clec9A Ab 10B4 using subcutaneous, intraperitoneal or intravenous (i.v.) routes, then measured the resulting anti-rat Ig response (Supplementary Fig. 1). As the responses were comparable, we proceeded with the i.v. route of administration. The targeting constructs carrying M2e or SP70 (Fig. 1a) were injected into C57BL/6 mice, at 2 μg per mouse, a level found to give maximal Ab titres with previous Ags.^7^ This dose was equivalent to 0.21 μg of M2e and 0.15 μg of SP70 Ag. The constructs were injected with or without CpG as a DC activation agent (Fig. 1b). The kinetics of the specific Ab responses were then followed (Fig. 1c, d).

One injection of the anti-Clec9A-M2e construct produced high and long-lasting IgG Ab titres even without the CpG adjuvant (Fig. 1c). The response to M2e was comparable to the simultaneously generated Ab response against the rat Ig (Fig. 1e). This strong response to M2e was clearly a consequence of targeting to Clec9A, as it was much higher than the response to the construct of M2e linked to the isotype-matched control mAb. The Clec9A-targeted response to M2e was only marginally increased by co-administration of CpG, in line with our previous experience targeting OVA and rat Ig to Clec9A.^7^ However, this high response to M2e was less than a “maximal” response produced in separate experiments by injecting C57BL/6 mice with 50 μg of M2e coupled to keyhole limpet haemocyanin (KLH) in complete Freund’s adjuvant then boosting with the same Ag in incomplete Freund’s adjuvant 2 weeks later (Fig. 1c; dotted line).

Targeting SP70 to Clec9A also produced a greatly elevated IgG Ab response compared with the isotype-matched control construct (Fig. 1d), but the level of the response was lower than that obtained with M2e. This was a feature of the Ag itself rather than a reduced targeting efficiency, as the internal control response to the rat Ig was high and similar to that obtained with the M2e construct (Fig. 1f). In contrast to the result with M2e, the targeted response to SP70 was substantially enhanced by co-administration of CpG (Fig. 1d).

### Mouse strain variation in the targeted responses

**Fig. 2:**
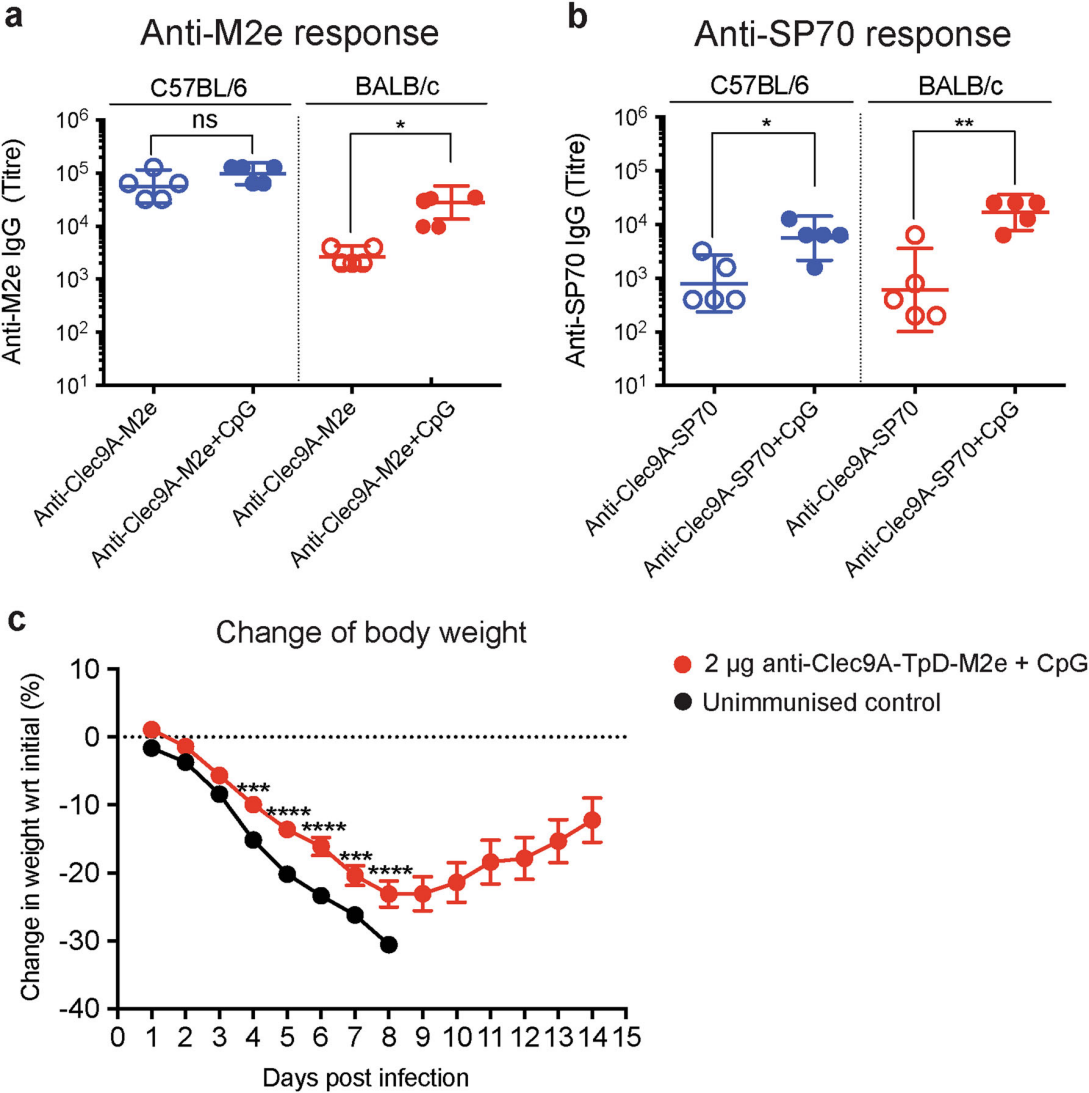
Responses to a single injection of Clec9A-targeted vaccine Ag: mouse strain variation, effect of adjuvant and resistance to infection. **a**, **b** Ab responses. Groups of C57BL/6 (*n* = 5) and BALB/c (*n* = 5) mice were immunised as in Fig. 1b. Serum samples were collected 2 weeks post immunisation and **a** the anti-M2e IgG response or **b** the anti-SP70 IgG response was measured by ELISA. Each point represents one individual mouse and the end point titre is shown as geometric mean with 95% CI. This side by side comparison was performed once but the individual strain responses were confirmed in multiple experiments. Data were analysed by an unpaired two-tailed Student's *t*-test, and significance was indicated as *p < 0.05, **p < 0.01. ns represents no significant differences. **c** Body weight following influenza infection. BALB/c mice (*n* = 10 mice per group) were immunised with 2 μg anti-Clec9A-TpD-M2e construct adjuvanted with 5 nmol of CpG (i.v.). An unimmunised control group was injected with 100 μl PBS. One month (30 days) later they were challenged by intratracheal administration of a lethal dose (400 PFU) of H1N1/PR8 virus. Body weight was recorded daily and monitored over a period of 14 days post infection. Mice were euthanised when they lost 30% or more of body weight. All mice in the unimmunised group were euthanised by day 8. One mouse in the immunised group was euthanised at day 8. Results are means with SEM. Statistical analysis was performed using two-way ANOVA analysis, followed by multiple comparisons with significance indicated as *** *p* < 0.001, **** *p* < 0.0001

The commonly used inbred mouse strains C57BL/6 (H-2^b^) and BALB/c (H-2^d^) are known to respond differently to Ags and infections with distinct polarisations of T-cell responses and differing protection efficiencies.^27,28^ We have found differences between these strains in the adjuvant-free responses to Clec9A-targeted Ags.^19^ To check if our findings with putative vaccine Ags would apply across these different mouse strains, a direct side-by-side comparison of the responses to the M2e and SP70 constructs was made (Fig. 2a, b). In the absence of adjuvant, the response of BALB/c mice to Clec9A-targeted Ags was lower than that of C57BL/6 mice. Whereas the co-administration of CpG gave only a small increase in the Ab titres of C57BL/6 mice, it produced a 10-fold increase in BALB/c mice. In the presence of the adjuvant, the responses of C57BL/6 and BALB/c mice to Clec9A-targeted M2e and SP70 were similar and both were high. We concluded an adjuvant would usually be required to maximise the Clec9A-targeted response. We compared CpG with alum and found CpG more effective for the Clec9A targeting (Supplementary Fig. 2). All further Ab response experiments involved using CpG as adjuvant and focused on the BALB/c mice used for our influenza infection model.

### Primary response to Clec9A-targeted Ag partially protect mice from influenza infection

It was important to determine whether the high anti-M2e Ab titres attained from Clec9A targeting could protect against viral infection. To this end, BALB/c mice were immunised with 2 μg of M2e coupled to the rat anti-Clec9A mAb 10B4, along with CpG as adjuvant. One month (30 days) later, mice were challenged with a high lethal dose (400 plaque-forming units (PFU)) of H1N1/PR8 virus, given intratracheally. All the non-vaccinated mice lost over 30% of their body weight and required euthanisation by day 8. By contrast, most mice (90%) immunised by targeting M2e to Clec9A survived the infection, although with substantial loss of body weight prior to recovery (Fig. 2c). In conclusion, targeting the weak Ag M2e to Clec9A provided significant protection from infection, but further boosting of the response was required for complete protection.

### Factors limiting a prime-boost approach

Although a single injection of the targeting construct may sometimes give Ab titres in a high and potentially protective range, in many cases a higher response would be needed. The standard approach to increasing Ab titres involves boosting the response with repeated injections. However, as shown in Fig. 1, a secondary injection boost with the targeting construct was ineffective, the response being no higher than a side by side primary response with the same construct dose. This applied regardless of whether the initial titre was high (M2e, Fig. 1c) or only moderate (SP70, Fig. 1d). It also applied when responses were elevated by the use of CpG as an adjuvant in both the priming and the boost (Supplementary Fig. 3). This reflected our findings with other Ags, including rat Ig, as confirmed in Fig. 1e, f. Why then did the otherwise efficient Clec9A targeting system fail as a boost injection?

**Fig. 3:**
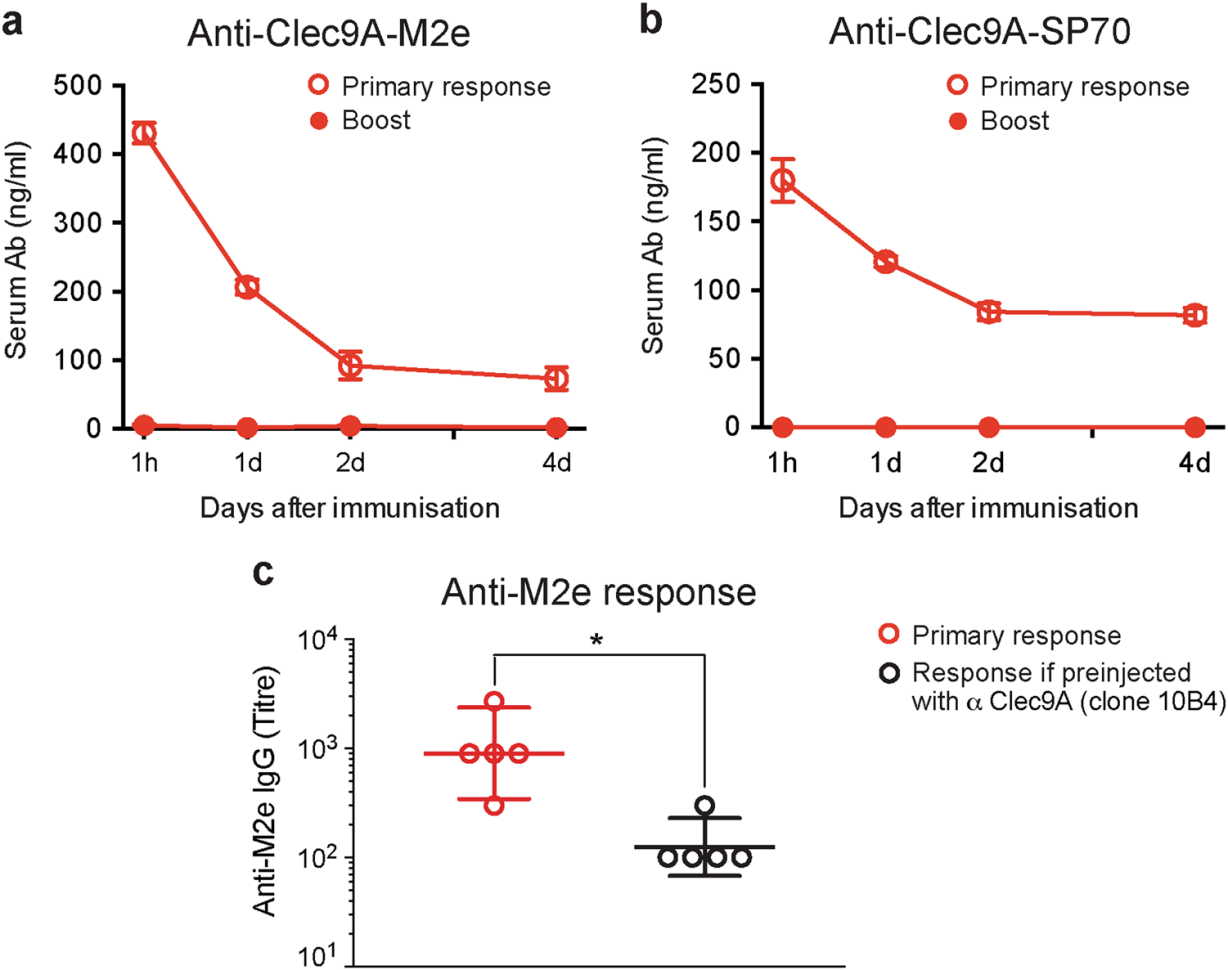
Effects of pre-injection with constructs on the persistence of the anti-Clec9A-Ag constructs in the serum and on Ab responses. **a**, **b** Serum persistence. C57BL/6 mice were injected i.v. with **a** 2 μg rat anti-Clec9A-M2e mAb construct or **b** 2 μg rat anti-Clec9A-SP70 mAb construct, both constructs being made using the rat mAb clone 10B4. At day 28 after priming, all groups of mice were given a second injection of 1 μg anti-Clec9A-M2e or 1 μg anti-Clec9A-SP70, respectively. Serum samples were collected at the indicated times post immunisation and the concentration of the rat Ig mAb in the serum was measured by ELISA. Open circles represent the values from the primary response, closed circles the values from the boost response. Each point represents pooled mean with SEM from five mice. **c** Ab responses following pre-injection with anti-Clec9A mAb only. BALB/c mice (*n* = 5 mice per group) were injected i.v. with 100 μl PBS for the primary response group or with 2 μg anti-Clec9A mAb (clone 10B4, not linked to M2e) for the Ab pre-injected group. Two weeks later, both groups of mice were given an injection of 2 μg anti-Clec9A-M2e mAb construct. A further 2 weeks later, serum samples were collected and the anti-M2e IgG response was measured by ELISA. Each point represents one individual mouse and the end point titre is shown as geometric mean with 95% CI. Results represent a single experiment. Data were analysed by an unpaired two-tailed Student's *t*-test, and significance was indicated as *p < 0.05

A likely reason for this failure was the high Ab titre against rat Ig of the targeting construct generated in the primary response (Fig. 1e, f). This could remove the targeting construct from the circulation, preventing the prolonged presentation required for efficient T_FH_ generation.^17^ Indeed, in separate studies (data not shown) this appeared to be the reason why constructs of OVA linked to rat anti-Clec9A failed to boost anti-OVA CTL responses. To determine if this was the likely reason for the boost failures in Fig. 1, we determined the relative levels of the M2e and SP70 targeting constructs in the serum after a primary or after a boost injection. As shown in Fig. 3a, b, the constructs persisted in the serum after the primary injection but were rapidly removed after a boost injection. To determine whether the serum Ab against the rat Ig itself was sufficient to block subsequent immune responses against Ag delivered using rat Ig-Ag constructs, we immunised either naive mice or mice pre-injected with the anti-Clec9A Ab 10B4, with the anti-Clec9A-M2e Ab construct (Fig. 3c). Mice that had been previously immunised with anti-Clec9A induced a poor response to M2e delivered using the anti-Clec9A-M2e construct. We concluded that for an effective boost, it would be essential to avoid the effects of Ab against the Ig of the targeting construct.

### Constructs suitable for a prime-boost strategy

To avoid any cross-reactivity between the prime and the boost anti-Clec9A Abs, a series of phage display Abs against mouse Clec9A were generated from a human Ig library by selection on the Clec9A extracellular domain.^29^ To reduce immunogenicity in mice, chimeric constructs were then made using these human V regions linked to mouse heavy and light chain C regions. Two of these chimeric Abs, those using clone 2 and clone 19 human V regions, bound specifically to Clec9A on the surface of mouse cDC1 as effectively as the rat anti-Clec9A mAb 10B4 (Supplementary Fig. 4).

**Fig. 4:**
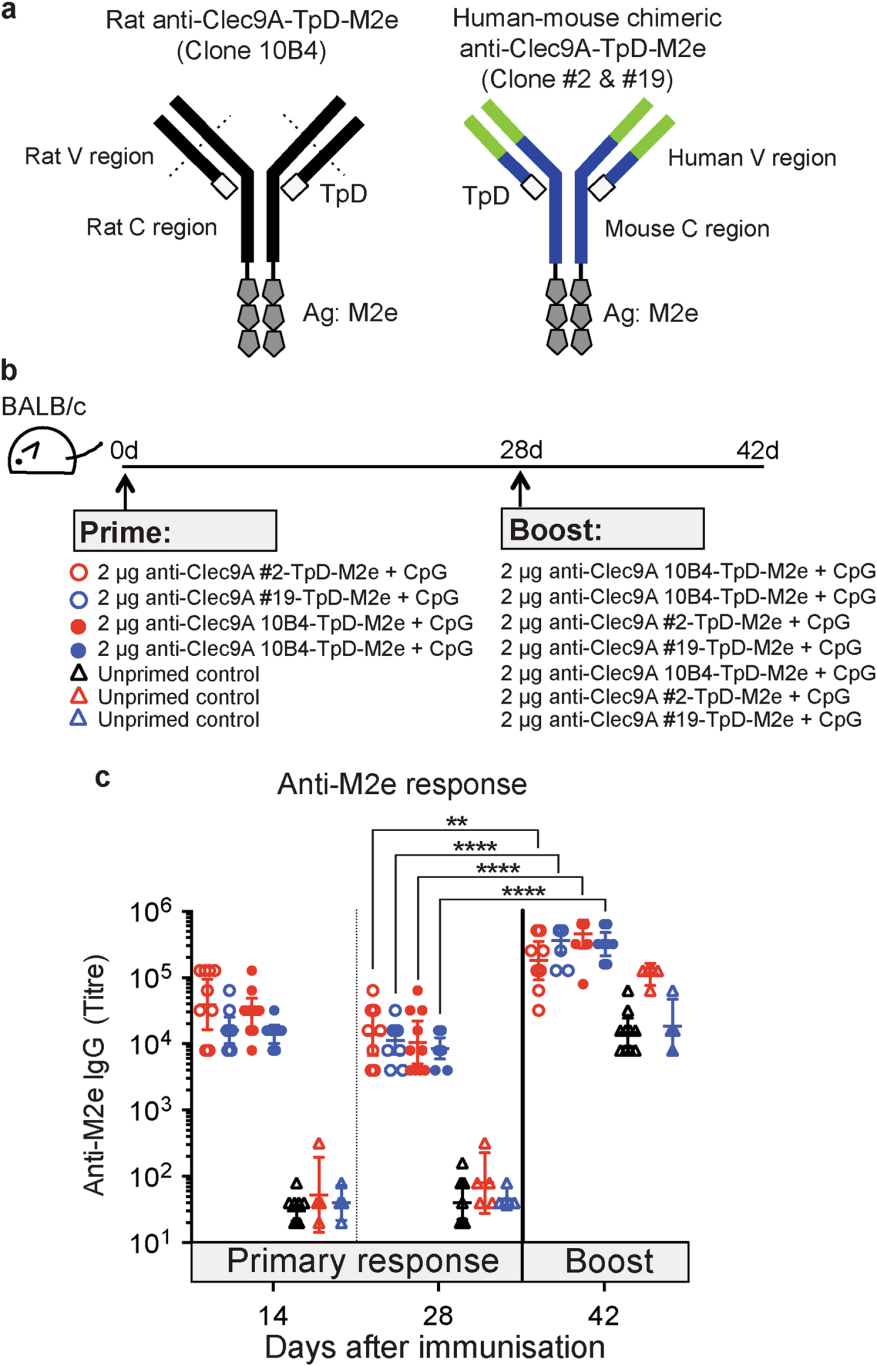
Priming and heterologous boosting of BALB/c mice using human–mouse chimeric anti-Clec9A Ab. **a** Schematic representation of the two new chimeric human–mouse anti-Clec9A-TpD-M2e constructs prepared from phage display Abs, compared with the rat anti-Clec9A-TpD-M2e construct. **b** Schematic representation of the experimental setup. BALB/c mice (*n* = 5 mice per group) were injected i.v. with 2 μg human–mouse chimeric anti-Clec9A-TpD-M2e (clones #2 or #19) or 2 μg rat anti-Clec9A-TpD-M2e (clone 10B4) in the presence of 5 nmol CpG. An unprimed control group was injected with 100 μl PBS. At day 28 after priming, each group of mice primed with the human–mouse chimeric Ab constructs were boosted with 2 μg rat anti-Clec9A-TpD-M2e (clone 10B4) and the groups of mice primed with the rat anti-Clec9A construct boosted with 2 μg human–mouse chimeric anti-Clec9A-TpD-M2e (clones #2 or 19) in the presence of 5 nmol CpG. Unprimed control groups were likewise injected at day 28 to give a primary response for direct comparison. **c** Serum samples were collected at the indicated time points post immunisation and the anti-M2e IgG response was measured by ELISA. Each point represents one individual mouse and the end point titre is shown as geometric mean with 95% CI. This experiment was performed twice and the results pooled (*n* = 10 mice per group). The boost responses were analysed by unpaired two-tailed Student's *t*-test and significance was indicated as ** *p* < 0.01, ***p < 0.001, ****p < 0.0001. The differences of Ab response between day 28 and at day 42 were indicated in Fig. 4c and the differences of Ab response between primed-boosted groups and unprimed-boosted groups at day 42 are followed; red open circle vs. black open triangle (**p = 0.0012), blue open circle vs. black open triangle (****p < 0.0001), red closed circle vs. red open triangle (***p = 0.0008), and blue closed circle vs. blue open triangle (**p = 0.0024)

Although such a heterologous prime-boost strategy should avoid the problem caused by Ab against the rat Ig, there may then be insufficient common T-cell epitopes for boosting the helper T-cell response. The T-cell epitopes in rat Ig that were crucial for the response to SP70 perhaps played a role in the response to M2e as well. To provide a common T-cell epitope for both the prime and the boost, we incorporated into the prime and the boost constructs the CD4 memory helper peptide TpD, known to induce potent recall responses in mice and primates.^30^ Genetically linked constructs were prepared with TpD linked to the light chain and M2e linked to the heavy chain of both the rat anti-Clec9A mAb 10B4 and the two hybrid human–mouse anti-Clec9A mAbs, as illustrated in Fig. 4a. The constructs were confirmed to bind efficiently to Clec9A on the surface of Clec9A transfectant cells (data not shown). In addition, we also prepared two non-targeting control constructs. In the first construct, M2e was linked to KLH, a protein known to provide effective T helper epitopes.^16^ In the second construct, M2e was linked to TpD helper epitope.

### Enhancing the Ab responses with a heterologous prime-boost strategy

We then used our new targeting constructs to ask if the already high primary response of mice to Clec9A-targeted M2e in the presence of CpG adjuvant could be further boosted by secondary injection of M2e linked to a Clec9A targeting Ab that should not cross-react with the primary targeting Ab (Fig. 4). The rat anti-Clec9A construct and the two chimeric human–mouse anti-Clec9A constructs gave similar primary anti-M2e responses. Priming with M2e linked to one anti-Clec9A targeting Ab followed by a secondary boost with M2e linked to an antigenically distinct anti-Clec9A Ab produced Ab titres that were above the primary response levels. The boosted responses were similar regardless of the order of primary and boost injections. We concluded that “heterologous” anti-Clec9A boosting is an effective strategy for obtaining enhanced responses.

The isotypes of the anti-M2e Abs produced in both the primary and boosted responses of Fig. 4 were determined (Supplementary Fig. 5). A range of Ig isotypes was produced, dominated by IgG1 and IgG2a. All anti-M2e Ig isotypes except IgM were increased on heterologous boosting. These results concur with other Ag responses using CpG as adjuvant.^31^

### Clec9A targeting as a priming strategy for a conventional non-targeted Ab response

**Fig. 5:**
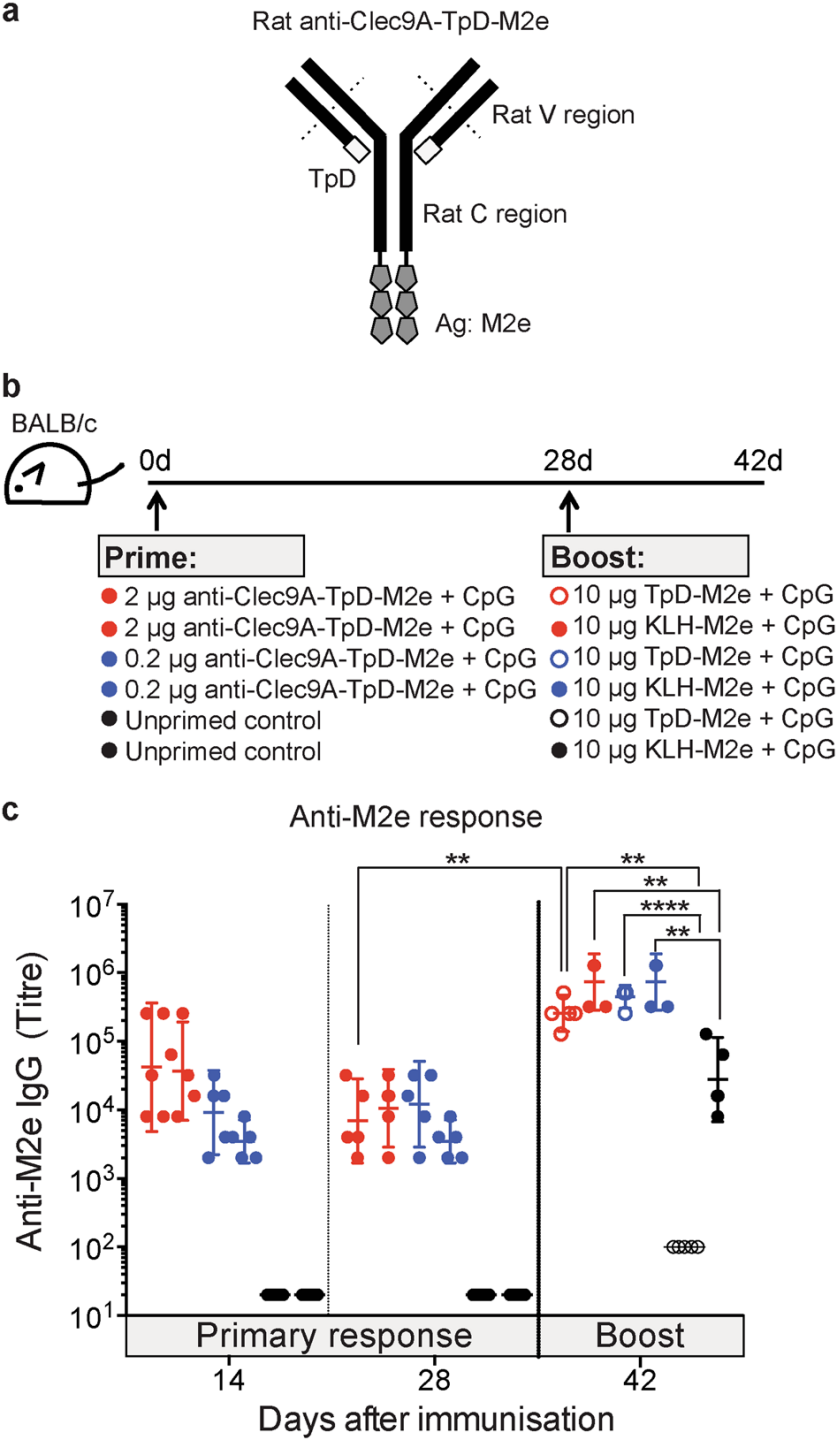
Ab responses generated by targeting M2e to Clec9A can be boosted using conventional, high-dose untargeted M2e complexes. **a** Schematic representation of the rat anti-Clec9A-TpD-M2e (clone 10B4) construct used for priming. **b** Schematic representation of the experimental setup. BALB/c mice (*n* = 5 mice per group) were injected i.v. with 0.2 μg or 2 μg anti-Clec9A-TpD-M2e in the presence of 5 nmol CpG. An unprimed control group was injected with 100 μl PBS. At day 28 after priming, each group of mice was boosted with either 10 μg of KLH-M2e or TpD-M2e, in the presence of 5 nmol CpG. **c** Serum samples were collected at the indicated times post immunisation and the anti-M2e IgG response was measured by ELISA. Each point represents one individual mouse and the end point titre is shown as geometric mean with 95% CI. Results represent a single experiment. The data with KLH-M2e boost were confirmed in a second experiment. Data were analysed by an unpaired two-tailed Student's *t*-test, and significance was indicated as **p < 0.01, ****p < 0.0001

We then asked if targeting of small amounts of Ag to Clec9A could serve to prime a conventional response to large amounts of untargeted Ag. This represented an alternative heterologous prime-boost strategy, but also served to check if a Clec9A-targeted priming immunisation was likely to improve the Ab response to a subsequent major infection. BALB/c mice were primed in the presence of CpG with an anti-Clec9A targeting construct that carried the M2e Ag on the heavy chain and the T-cell epitope TpD on the light chain (Fig. 5a). Priming doses of 2 or 0.2 μg of the construct were compared, corresponding to 0.2 and 0.02 μg of M2e, respectively. After 28 days, the mice were boosted with 10 μg of M2e linked to KLH, or 10 μg of M2e linked to TpD, again with CpG as adjuvant (Fig. 5b). The boost thus represented a 50-fold or 500-fold higher dose of the M2e Ag compared with the priming doses.

Clec9A-targeted priming with M2e significantly increased the untargeted response to M2e linked to KLH (Fig. 5c), and strikingly increased the untargeted response of M2e linked to TpD (Fig. 5c). Although the primary response to 0.2 μg of M2e linked to anti-Clec9A mAb was lower than the primary response to 2 μg, the lower dose was equally effective at priming for the subsequent boost. It was concluded that targeting Ag to Clec9A is an effective priming strategy that can enhance the response to conventional untargeted Ag vaccination.

The converse approach of priming with untargeted M2e linked to KLH, then boosting by targeting M2e to Clec9A, also gave an effective boost to the Ab titres (Supplementary Fig. 6). This suggests Clec9A targeting could also serve to improve the immune response of an already immunised population.

### M2e immunisation by Clec9A-targeted priming followed by conventional untargeted boosting fully protects mice from influenza infection

**Fig. 6:**
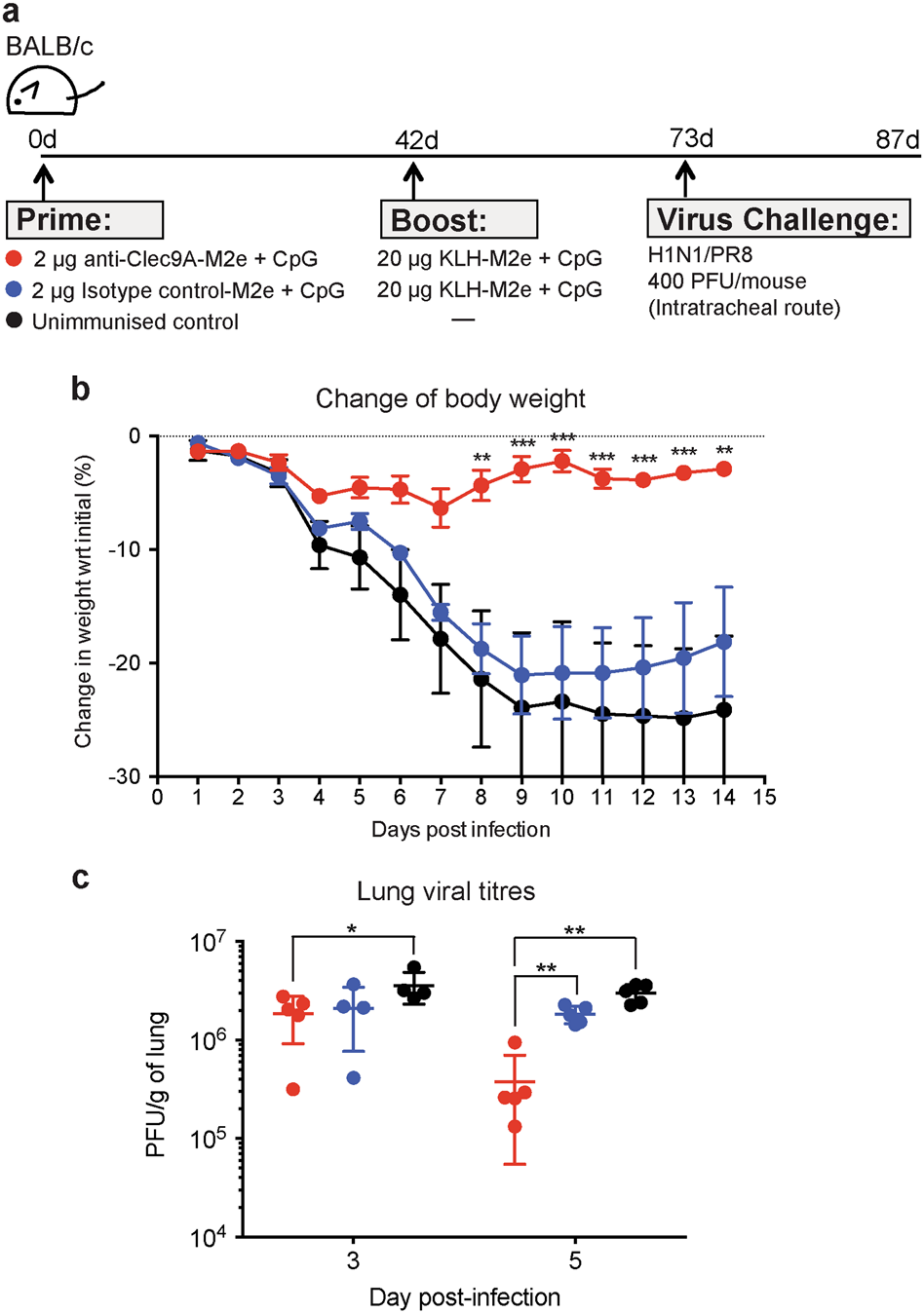
Immunisation with M2e via Clec9A-targeted priming then conventional boosting protects mice against lethal influenza challenge. **a** Schematic representation of the experimental setup. BALB/c mice (*n* = 8–10 mice per group) were primed with 2 μg anti-Clec9A-M2e construct or the non-targeting isotype control-M2e construct, adjuvanted with 5 nmol of CpG (i.v.), then boosted 42 days later with 20 μg M2e-KLH adjuvanted with 2.5 nmol of CpG accordingly to the schedule shown. An unimmunised control group was injected with 100 μl PBS. One month (30–31 days) later they were challenged by intratracheal administration of a lethal dose (400 PFU) of H1N1/PR8 virus. **b** Body weight following influenza infection. Body weight was recorded daily and monitored over a period of 14 days post infection. Mice that lost 30% or more of their body weight were euthanised; by day 8 this amounted to 60% of the unimmunised group, 20% of the isotype control primed group, but none of the Clec9A-targeted primed group. Results are means with SEM of the surviving mice. Statistical analysis was performed using two-way ANOVA. (anti-Clec9A-M2e + CpG vs. Isotype control-M2e + CpG), and significance was indicated as **p < 0.01, ***p < 0.001. This result represents a single experiment, but two additional experiments gave equivalent protection results. **c** Lung viral titres. Lungs were harvested on days 3 and 5 post challenge with influenza virus. Influenza virus titres were determined by the plaque assay. Results represent a single experiment, initiated with *n* = 5 mice per group. However, one mouse of the unimmunised control group and one mouse of the isotype control group died by day 3; all targeted mice survived. Error bars represent mean with SD of surviving mice. Data were analysed by Mann–Whitney test and, significance was indicated as *p < 0.05, **p < 0.01

It was important to determine if the higher Ab titres attained from the prime-boost strategy of Fig. 5 would be associated with improved protection against a viral infection (Fig. 6a). Therefore, BALB/c mice were primed with 2 μg of M2e coupled to the rat anti-Clec9A mAb 10B4 along with CpG as adjuvant. They were boosted at 42 days with 20 μg of M2e coupled to KLH, along with CpG. One month after the boost, they were challenged with a high lethal dose (400 PFU) of H1N1/PR8 virus, given intratracheally.

All mice primed by targeting M2e to Clec9A survived the infection, with only a minimal transient body weight loss (Fig. 6b). This correlated with significantly reduced lung viral titres on day 5 post infection (Fig. 6c). By contrast, non-vaccinated mice all showed marked body weight loss and 60% reached the euthanasia end point (Fig. 6). Importantly, mice immunised with M2e linked to the non-targeting isotype control mAb also showed marked body weight loss, which led to euthanasia of 20% of the animals. In conclusion, targeting the weak but “universal” Ag M2e to Clec9A primed immunity, which upon boosting resulted in a strong protective response against influenza virus.

## Discussion

A major objective of this project was to assess if targeting putative vaccine Ags to Clec9A on the cDC1 subset of DCs would produce the same marked enhancement in Ab responses as seen with model Ags such as OVA^7,17^ or NP linked to a suitable carrier protein.^16^ The model Ags used by immunologists have been selected to give good responses, and accordingly they contain effective B- and T-cell epitopes for which the test mice have an adequate frequency of responsive T and B cells even before priming. This is not necessarily the case with any candidate vaccine Ag. Targeting the putative vaccine Ags considered here to Clec9A produced primary responses covering a range we have seen with other vaccine candidates. Targeting M2e to Clec9A gave high Ab responses approaching that of our previous model Ags. In contrast to M2e, targeting SP70 to Clec9A gave a low but still positive response. Importantly, the Clec9A targeting strategy clearly worked with both vaccine candidates as the response to injection of tiny amounts of the Ag linked to anti-Clec9A mAb was much higher than the response to Ag linked to an isotype-matched control mAb. Much of the project then involved strategies to enhance, when needed, the initial primary responses to a level likely to be protective.

One obvious strategy was the use of an appropriate adjuvant. A surprising feature of the Clec9A-targeted response to our previous model Ags was that high Ab responses could be obtained in the absence of adjuvants or any sign of DC activation, suggesting the side effects of adjuvants might be avoided in a Clec9A-targeted vaccine.^2,7^ The high response without adjuvants was shown to be in part due to the persistence of the Clec9A targeting constructs in the bloodstream, leading to prolonged Ag presentation even by non-activated DCs, leading in turn to extended T_FH_ production.^17,21^ However, even in this situation the production of T_FH_ was clearly greater when the DC activation agent CpG was administered,^17^ and this increase in T_fh_ could be important with weak Ags. In the present study, targeting M2e to Clec9A in C57BL/6 mice gave a high Ab response that was only marginally enhanced by co-injection of CpG, much as seen with our previous model Ags. However, the same approach in BALB/c mice indicated a requirement for the adjuvant for a maximal response, and a clear requirement for adjuvant was apparent with the SP70 Ag in both mouse strains. Accordingly it seems likely that a DC-activating adjuvant would need to be a component of a Clec9A-targeted vaccine. It is possible that the amount of adjuvant could be reduced with Clec9A targeting, and simultaneous targeting of the Ag and the DC-activating agent to the cDC1 subset is a possible future strategy for reducing the side effects of generalised innate immune system activation.

Our results with targeting M2e and SP70 to Clec9A suggest that a single injection of targeted Ag, even with adjuvant, might not be adequate for efficient immunisation. Although a single injection of Clec9A-targeted M2e gave high Ab responses, this was not the case with SP70, even when CpG as adjuvant was used. In our experience, some other vaccine candidate Ags present the same problem. In addition, although the single injection of M2e targeted to Clec9A allowed most mice to survive a lethal dose of influenza, there was still a marked weight loss indicating incomplete protection. The usual solution to this problem is to give multiple injections, or at least two injections in a prime-boost sequence, to expand to level of responding B and T cells. However, our initial attempts to both prime and boost the response using Clec9A targeting failed, the boosted response being no higher than a primary response. As we consider this to be a basic problem with Ab-based targeting, we have explored the reason for this failure and devised several strategies to improve the prime-boost response.

The reason for the failure to obtain an effective boost appeared to be the same as we have already shown for Clec9A-targeted responses to OVA (data not shown), namely that the initial high Ab response to the injected construct rapidly removed it from the bloodstream, preventing the prolonged Ag presentation that is one of the key advantages of Clec9A targeting. Note that this was mainly caused by the high Ab response to the foreign rat Ig of the anti-Clec9A mAb, rather than the lower primary response to the vaccine Ag itself. Although a very high response to a vaccine Ag would presumably present the same problem, a secondary boost would not be required if the primary response was that effective. The ideal strategy to avoid Ab responses to the rat anti-Clec9A targeting Ab would be to use a second targeting construct that shared negligible cross-reactivity. Thus, we designed a “heterologous” prime-boost strategy, using the rat anti-Clec9A mAb 10B4 for the primary injection, and a modified phage display generated human–mouse chimeric anti-Clec9A for the boost, or visa versa. The advantages of heterologous prime-boost systems have been noted in other contexts.^32–34^ This approach allowed an effective boost of the Ab response to M2e. Although fully humanising anti-CLEC9A Abs, such as has been accomplished for the anti-human DEC205 Abs,^5,6^ should solve such boosting problems for human application, a heterologous targeting strategy may offer further advantages.

An alternative strategy for improving the Ab response in a prime-boost sequence is to use Clec9A targeting only for the priming step, and boost in a more conventional way, such as with larger quantities of Ag linked to a suitable carrier. This has the advantage that even lower (sub-microgram) amounts of the targeting construct are needed for priming compared with generating a strong primary response. This has worked well for us with model Ags such as OVA, and we now find it effectively boosts the response to M2e. Priming the response system by targeting tiny doses of Ag to CLEC9A may improve the outcome of many standard human vaccination strategies where the primary response has been inadequate.

It is particularly important that Clec9A-targeted priming with the normally weak Ag M2e, followed by a single “conventional” boost with M2e coupled to a carrier, served to effectively immunise mice and provide full protection against influenza infection. Multiple injections are normally required to produce a significant response to M2e. As M2e is a possible “universal” Ag for common influenza strains, application of this approach to human populations could reduce the need for yearly immunisation against influenza. Currently, we are involved in optimising the adjuvant, Ag dose and administration requirements for Clec9A-targeted vaccines against influenza, EV71 and dengue viral infections. Such detailed studies will guide the application of CLEC9A targeting to human vaccine applications. Although we have emphasised the role of Abs in the initial protection against viral infections, Clec9A targeting of Ags in the presence of adjuvants such as adopted here is also a very effective procedure for generating CTL.^9,17^ The vaccine Ags we have used here do not appear to have effective CD8 T-cell epitopes for mice,^23,25^ but in future human applications targeting vaccine Ag with CD8 T-cell epitopes to CLEC9A could further improve virus clearance.

## Materials and methods

### Mice

The mice used for Ab production studies were 8-week-old C57BL/6 and BALB/c females. For DC-binding studies, these mice were compared with Clec9A KO mice.^18^ The mice were bred and maintained under specific pathogen-free (SPF) conditions at the Walter and Eliza Hall Institute (WEHI). For the influenza infection studies, 7- to 8-week-old female Taconic BALB/c mice (InVivos), of Restricted Flora status and housed under SPF conditions, were used. Mice were randomly assigned to different immunisation groups. The investigators were not blinded to analysis.

### Anti-Clec9A Abs used in targeting constructs

The initial targeting constructs were based on the rat IgG2a anti-mouse Clec9A mAb 24/04-10B4 and used as control the non-targeting isotype control rat IgG2a GL117, which recognises *E.coli* β-galactosidase.^7,17,35^ To obtain anti-mouse Clec9A Abs that were antigenically distinct, human IgG1-based anti-Clec9A Abs were generated by phage display from a human Ig library.^29^ The phage display Ab were selected by binding to the extracellular domain of Clec9A. They were initially screened on the basis of binding to Clec9A by enzyme-linked immunosorbent assay (ELISA), and finally selected for strong binding to the surface of Clec9A-transfected cells as visualised by flow cytometry.

### Generation of Clec9A targeting constructs by genetic fusion of anti-Clec9A Abs with Ags

Plasmids encoding recombinant Ab heavy and light chains in pcDNA3.1+ were generated by gene synthesis of codon-optimised variable region sequences in frame with either the rat IgG2a^17^ or mouse IgG1 constant regions as previously described (GeneArt).^36^ Ab constructs carrying the M2e vaccine candidate were generated by fusing the Ab heavy chain to antigenic sequence (MAAAMSLLTEVETPIRNEWGCRCNDSSDGGGMSLLTEVETPIRNEWGCRCNDSSDGGGMSLLTEVETPIRNEWGCRCNDSSDGGG) containing three tandem repeats of the M2e sequence MSLLTEVETPIRNEWGCRCNDSSD separated by a glycine linker. Similarly, Ab constructs carrying the SP70 vaccine candidate were generated by fusing the Ab heavy chain to antigenic sequence (MAAAYPTFGEHKQEKDLEYCGGGYPTFGEHKQEKDLEYCGGGYPTFGEHKQEKDLEYCGGG) containing three tandem repeats of the SP70 sequence YPTFGEHKQEKDLEYC separated by glycine linker. Ab constructs carrying the TpD sequence were generated by fusing the Ab light chain to the TpD sequence (AAAAILMQYIKANSKFIGIPMGLPQSIALSSLMVAQ).^30^ Thus, recombinant Ab–Ag sequences carried 6 Ag per Ab molecule (three copies per heavy chain) and where required they carried 2 TpD sequences per Ab molecule (one copy per light chain). Ab–Ag were expressed in Freestyle 293F cells (Life Technologies) using 293Fectin (Invitrogen) and purified from culture supernatants using protein G as previously described.^17^

### Immunisation for Ab production using anti-Clec9A-Ag constructs

Mice were injected i.v. with specified amounts (usually 2 μg) of targeting constructs consisting of Ag genetically fused anti-Clec9A Abs (anti-Clec9A 10B4-Ag, anti-Clec9A 10B4-TpD-Ag, anti-Clec9A #2-TpD-Ag or anti-Clec9A #19-TpD-Ag, or isotype control GL117-Ag) in the absence or presence of 5 nmol synthetic phosphorothioate CpG-1668 (GeneWorks), all in 100 μl sterile phosphate-buffered saline (PBS). For some boosting experiments, the mice were injected with 10–20 μg M2e Ag linked to either KLH or TpD (Mimotopes, Australia). To obtain an estimate of a maximal Ab response, a group of C57BL/6 mice were injected with 50 μg KLH-Ag with Freund’s complete adjuvant and 2 weeks later, the mice were boosted with 50 μg KLH-Ag with Freund’s incomplete adjuvant, and blood collected at day 30 after the boost. For negative or unprimed control groups, mice were i.v. injected with 100 μl PBS.

### Measurement of Ab responses by ELISA

ELISA assays for Abs against the pathogen Ags was carried out as previously described.^7^ Briefly, round-bottom 96-well ELISA microtiter plates (Costar) were coated at 4 **°**C overnight with 1–3 μg/ml KLH or OVA-conjugated M2e or SP70 peptide (Mimotopes) for specific Ag Ab responses, or with 1.5 μg/ml rat GL117 mAb for anti-rat Ig Ab responses. Unbound peptide or protein was washed away (PBS, 0.05% Tween 20) and serially diluted serum samples (PBS, 5% milk powder) were plated and incubated at 4 **°**C overnight. Bound mouse IgG Abs was detected using donkey anti-mouse IgG-HRP (Millipore, catalogue # AP192P) and visualised using ABTS substrate (Sigma). Optical density (OD) was measured at 405–490 nm. End point titres were calculated by using cut-off values defined as at least five times the OD of a no-serum negative control. To determine the level of Ig isotypes, bound mouse IgG1 (catalogue # 1070-05), IgG2a (catalogue # 1080-05), IgG2b (catalogue # 1090-05), IgG3 (catalogue # 1100-05) and IgM (catalogue # 1021-05) were detected using goat anti-mouse isotype Ig HRP (SouthernBiotech).

### Measurement of in vivo persistence of anti-Clec9A constructs

Serum samples were obtained from C57BL/6 mice injected once with 2 μg of the anti-Clec9A-M2e Ab construct or the anti-Clec9A-SP70 Ab construct, and from mice primed and then boosted 28 days later with the same constructs. The ELISA assay was as previously described.^19^ Briefly, ELISA plates were coated with 0.1 μg/ml mouse Clec9A at 4 **°**C overnight. Unbound protein was washed away before plates were blocked with 1% BSA/PBS for 30 min at room temperature. Serially diluted serum samples were then plated and incubated for 2 h at room temperature. Bound Ab were detected with anti-rat IgG2a biotin (BD Pharmingen, catalogue # 553894) for 1 h at room temperature, followed by SA-HRP (GE Healthcare, catalogue # RPN4401V) for 1 h at room temperature, then visualised with ABTS substrate. Each plate contained an internal standard curve generated using the anti-Clec9A-M2e construct or the anti-Clec9A-SP70 construct. The concentration of serum anti-Clec9A Ab was calculated based on this internal standard curve.

### Isolation and analysis of Clec9A on mouse splenic DCs

Spleen DCs were isolated and enriched as described previously.^37^ The isolated cDCs were stained with the following fluorochrome-conjugated mAbs: anti-CD11c (N418-FITC), anti-CD8 (YTS 169.4-APC), isotype control IgG2a biotin (eBioscience, catalogue # 13-4321-85), anti-Clec9A (10B4, #2, #19-biotin). Biotin was detected with SA-PE (BD Pharmingen, catalogue #554061) and nonspecific binding was blocked with pre-incubation of anti-CD16/32 (2.4G2) with 1% normal rat serum. The expression of Clec9A was determined on PI negative live CD8^+^ DC (CD11c^+^CD8^+^). Analysis was performed on a Fortessa instrument (Becton Dickinson).

### Assays for protection of mice from influenza infection

BALB/c mice (*n* = 8–10 mice per group) were administered i.v. with 2 µg of the rat anti-Clec9A-M2e 10B4 construct or isotype control GL117-M2e construct adjuvanted with 5 nmol of Class B CpG-1668 (Invivogen, catalogue # tlrl-1668) in a total volume of 100 µl in sterile PBS. Mice were boosted (i.v.) 42 days later with 20 µg of KLH-M2e (Mimotopes) adjuvanted with 2.5 nmol CpG. A third test group was injected with 100 µl PBS. Influenza challenge was performed 30–31 days post boost by intratracheal administration of 400 PFU of H1N1/PR8 virus. In most experiments, body weight was recorded daily, and any mice losing 30% or more of initial weight were euthanised. The weight of survivors was monitored over a period of 14 days post infection. To monitor the viral titre, lungs were harvested from euthanised mice 3 or 5 days post infection and stored at −80 °C before plaque assay.

### Plaque assay for influenza virus levels

The frozen lungs were thawed, weighed and homogenised in 2 ml PBS. The homogenates were clarified by centrifugation (10,000 rpm for 10 min at 4 °C) passed through a 0.44 µm syringe-driven filter unit, then 10-fold serially diluted in minimal essential medium (MEM, Life Technologies) supplemented with 2 µg/ml bovine pancreas TPCK-Trypsin. MDCK cells (1.5 × 10^5^ cells per well) were seeded onto 24-well plates and infected with 100 μl of the diluted lung homogenates for 1 h at 35 °C. The culture supernatants were then removed and 1 ml of 1.2% w/v Avicel (FMC BioPolymer) in MEM supplemented with 2 µg/ml bovine pancreas TPCK-Trypsin, 1.5% sodium bicarbonate and 1% HEPES was added as overlay media. After 3-day incubation at 35 °C, the cells were fixed with 4% formaldehyde for 1 h prior to washing with PBS and staining with crystal violet (Sigma-Aldrich). Upon removal of crystal violet via washing with PBS, plaques in each well were visually scored and the viral titres were expressed as PFUs per gram of lung tissue (PFU/g).

### Statistics

For animal studies, samples size with adequate statistical power was determined based on previous experiments. Statistical analysis was performed using Prism software (GraphPad software). A two-tailed unpaired Student's* t*-test was applied, unless otherwise indicated. No samples or animals were excluded from the analysis. The p-values are indicated as follows: *p < 0.05, **p < 0.01, ***p < 0.001 and ****p < 0.0001. ns represents no significant differences.

### Study approvals

Animal experimental procedures (Australia) were approved by the Animal Ethics Committee, WEHI, Parkville, Australia. Animal experiments (Singapore) were carried out in accordance with the guidelines of the National Advisory Committee for Laboratory Animal Research (NACLAR). Animal facilities are licensed by the regulatory body Agri-Food and Veterinary Authority of Singapore (AVA). The described animal experiments were approved by the Institutional Animal Care and Use Committee (IACUC) from National University of Singapore (NUS) under the protocol number R2013-04447.

### Data availability

The data that support the findings of this study are available from the corresponding author upon reasonable request.

## Electronic supplementary material

Supplemental Material
