# Supplementary material for: Enhancing vaccine antibody responses by targeting Clec9A on dendritic cells
Source: NPJ Vaccines. 2017 Nov 6;2:31. doi: 10.1038/s41541-017-0033-5 (PMC5674066; doi:10.1038/s41541-017-0033-5)
Supplement: Supplementary file 1 — Supplemental Material [file 41541_2017_33_MOESM1_ESM.pdf]

SUPPLEMENTARY INFORMATION

Supplementary Figure 1. Targeting Clec9A by different immunisation routes generates comparable Ab responses. C57BL/6 mice (n=5 mice per group) were injected with 10 µg anti-Clec9A mAb (clone 10B4) or 10 µg Isotype control mAb (clone GL117) by different routes (s.c.;subcutaneous, i.v.;intravenous and i.p.;intraperitoneal). Serum anti-rat Ig reactivity at d 14 post immunisation was measured by ELISA. Each point represents one individual mouse and the end point titre is shown as a geometric mean.

Supplementary Figure 2. Targeting vaccine Ag to Clec9A in the presence of adjuvants generates enhanced anti-SP70 Ab responses. BALB/c mice (n=5 mice per group) were injected with 2 µg anti-Clec9A-SP70 mAb construct, or 2 µg isotype control-SP70 mAb construct in the absence or presence of adjuvants, 5 nmol CpG-1668 (i.v.) or alum (i.p.). Serum samples were collected at d 14 to 28 post immunisation and anti-SP70 IgG response was measured by ELISA. Each point represents one individual mouse and the end point titre is shown as geometric mean with 95% CI. Results represent a single experiment but the results were confirmed in other experiments. Data was analysed by an unpaired two-tailed Student *t* test, and significance is indicated as $*p<0.05$, $** p<0.01$.

Supplementary Figure 3. Homologous Clec9A-targeted priming and boosting, in the presence of CpG adjuvant, does not significantly enhance Ab responses to M2e compared with Clec9A-targeted priming alone. (a) Schematic representation of the experimental setup. C57BL/6 mice (n=5 mice per group) were primed with 2 µg anti-Clec9A-TpD-M2e construct in the presence of 5 nmol CpG (i.v.) and unprimed control group was injected 100 µl PBS. At d 28 after priming, both groups of mice were boosted with same construct in the presence of 5 nmol CpG. (b) Serum samples were collected 14 d after the primary injection or after the boost injection and the anti-M2e IgG response was measured by ELISA. Each point represents one individual mouse and the end point titre is shown as geometric mean with 95% CI. This experiment was performed once. Data was analysed by an unpaired two-tailed Student *t* test. ns represents no significant differences.

Supplementary Figure 4. Conventionally generated and phage-display generated anti-Clec9A mAbs selectively bind to cell surface Clec9A expressed on CD8^{+} splenic cDC. Splenic cDCs were isolated from naïve C57BL/6 mice, from Clec9A KO mice on a C57BL/6 background, and from naïve BALB/c mice. The DCs were surface immunofluorescence labeled for CD11c (N418-FITC), CD8 (YTS 169.4-APC) and Clec9A, using the phage-display generated clones 2 and 19, and the conventionally generated rat mAb clone 10B4. The anti-Clec9A mAbs were biotinylated and Clec9A was detected using SA-PE. On flow cytometric analysis the CD8^{+} cDC (CD11c^{+}CD8^{+}) were gated and analysed for Clec9A fluorescence. The solid line represents Clec9A staining and the dotted line represents the background omitting the anti-Clec9A mAb.

Supplementary Figure 5. Isotypes of anti-M2e Abs following heterologous priming and boosting using different anti-Clec9A-M2e constructs. (a) Schematic representation of the experimental setup. BALB/c mice (n=5 mice per group) were immunised as in Figure 4. Serum samples were collected at the indicated time points post immunisation and (b-f) the isotypes (IgM, IgG3, IgG1, IgG2a, and IgG2b) of the anti-M2e Abs were measured by ELISA. Each point represents one individual mouse and the end point titre is shown as geometric mean with 95% CI. This experiment was performed once (n=5 mice per group). The differences due to boosting were analysed by unpaired two-tailed Student *t* test and significance is indicated as * $p<0.5$, ** $p<0.01$.

Supplementary Figure 6. Targeting Ag to Clec9A can boost Ab responses to free, untargeted Ag. (a) Schematic representation of the experimental setup. BALB/c mice (n=5 mice per group) were injected i.v. with 10 µg KLH-M2e in the absence or presence of 5 nmol CpG, or with 100 µl PBS for the unprimed control group. At d 28 after priming, all groups of mice were injected with 2 µg anti-Clec9A-TpD-M2e construct in the presence of 5 nmol CpG. (b) Serum samples were collected at the indicated times post immunisation and anti-M2e IgG responses were measured by ELISA. Each point represents one individual mouse and the end point titre is shown as geometric mean with 95% CI. This experiment was performed twice and pooled

data are shown (n=10 mice per group). Data was analysed by an unpaired two-tailed Student *t* test, and significance was indicated as $^{**}p<0.01$.

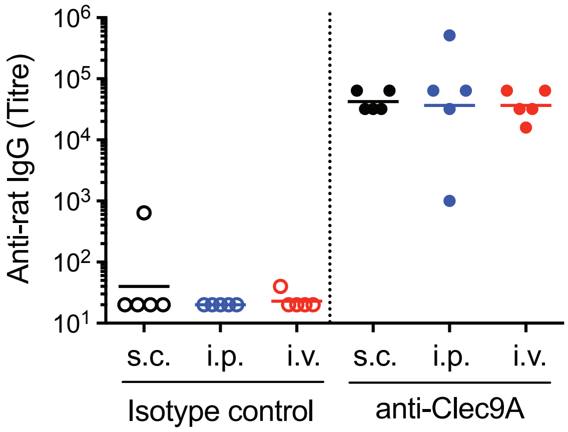

Supplementary Figure 1

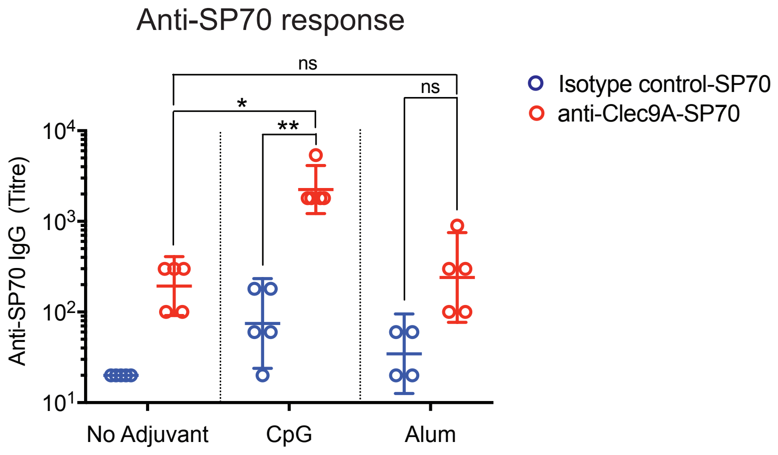

Supplementary Figure 2

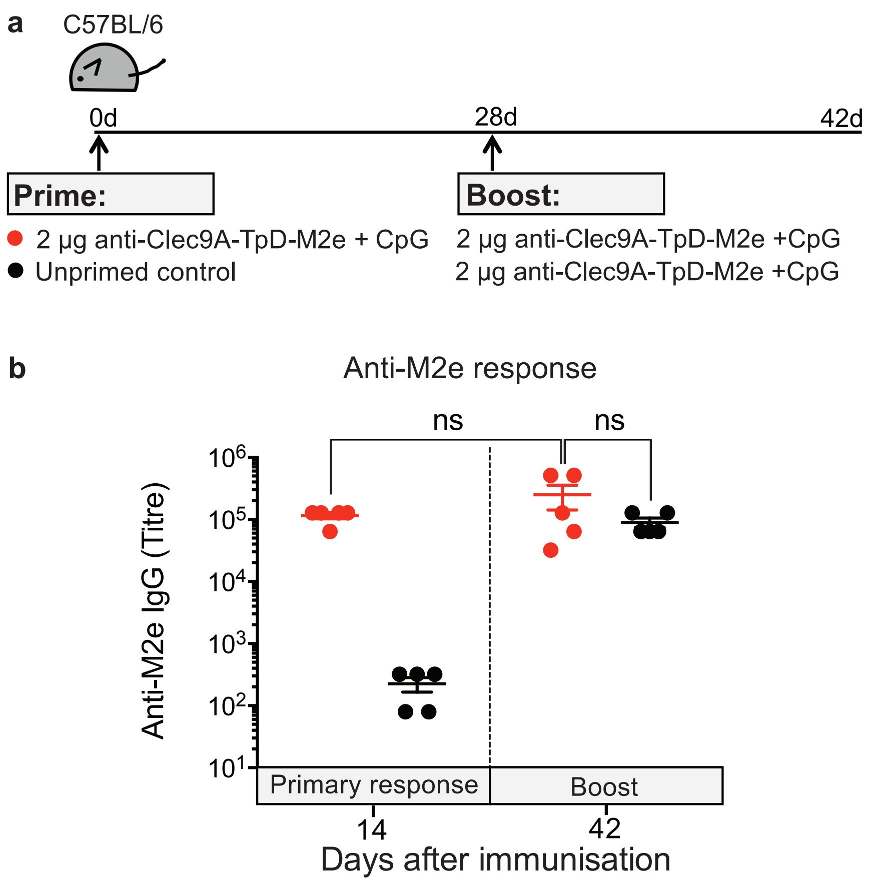

Supplementary Figure 3

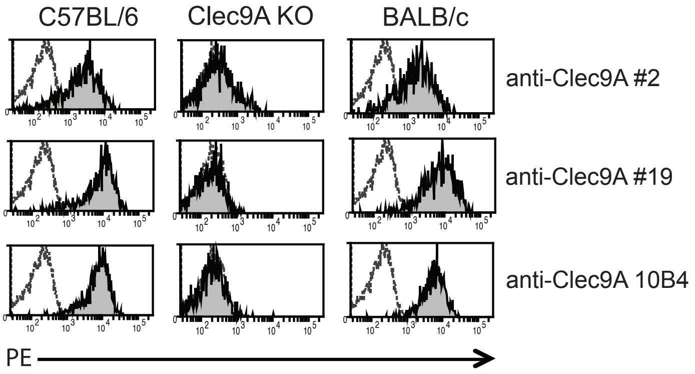

Supplementary Figure 4

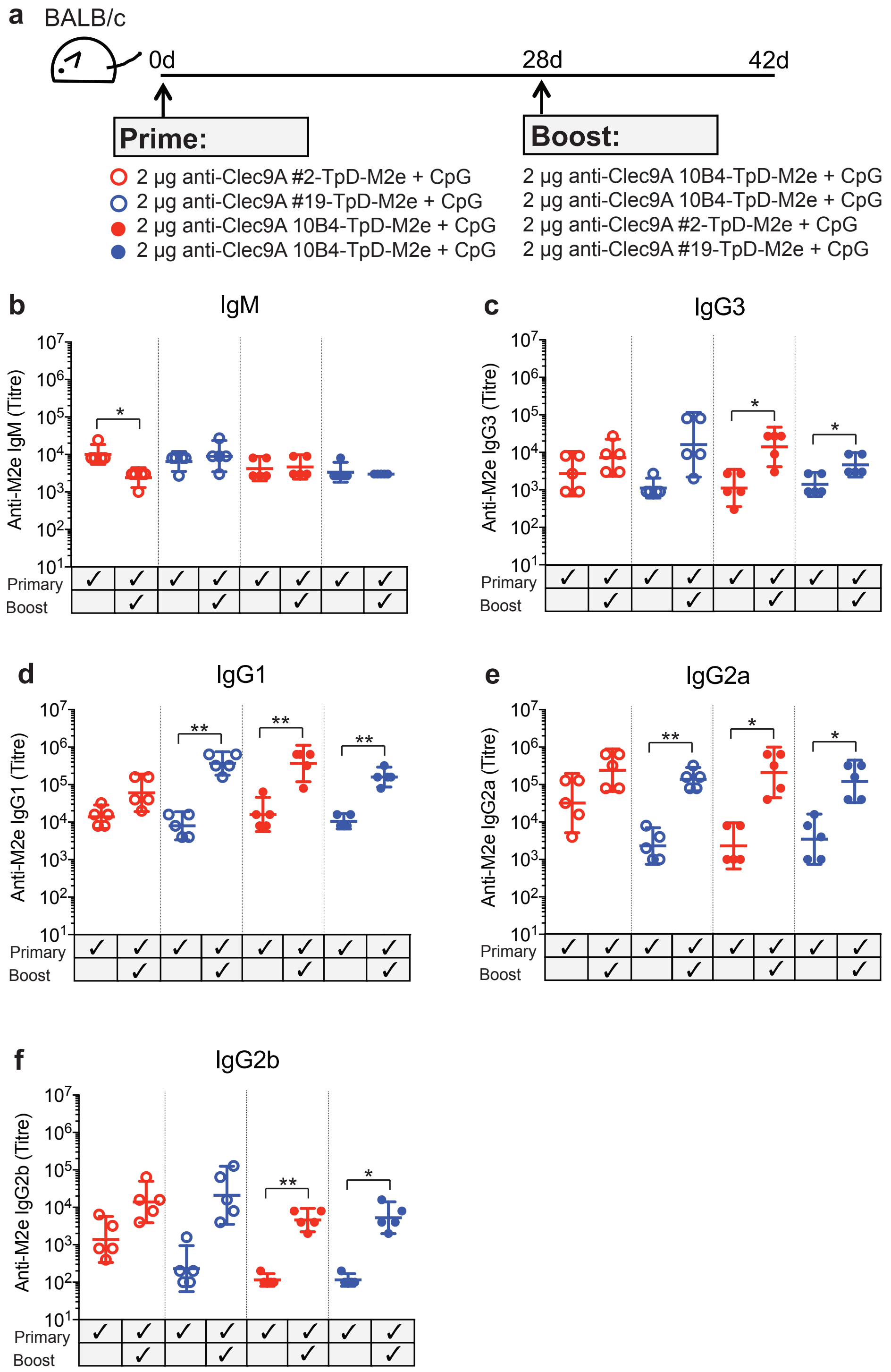

Supplementary Figure 5

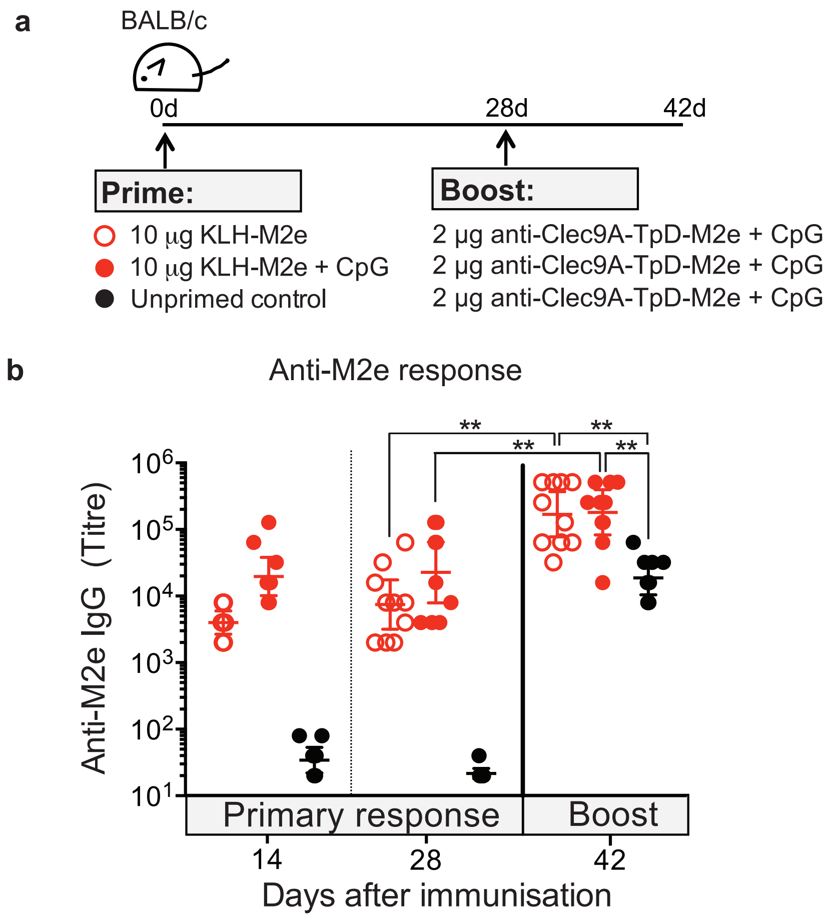

Supplementary Figure 6
